# Characterizing the fecal microbiota of infants with botulism

**DOI:** 10.1186/s40168-015-0119-0

**Published:** 2015-11-23

**Authors:** T. Brian Shirey, Janet K. Dykes, Carolina Lúquez, Susan E. Maslanka, Brian H. Raphael

**Affiliations:** Enteric Diseases Laboratory Branch, Centers for Disease Control and Prevention, Atlanta, GA 30329 USA

**Keywords:** Infant botulism, Fecal microbiota, Intestinal colonization, *Clostridium botulinum*, Botulinum neurotoxin

## Abstract

**Background:**

Infant botulism is the most prevalent form of botulism in the USA, representing 68.5 % of cases reported from 2001–2012. Infant botulism results when botulinum toxin-producing clostridia (BTPC) colonize the infant gut with concomitant in vivo production of the highly potent botulinum neurotoxin (BoNT). The gut microbiota of infants with botulism is largely uncharacterized; therefore, it remains unclear whether the microbiota profile of these patients are distinct in composition, abundance, or diversity. To address this uncertainty, we employed 16S rRNA gene profiling to characterize the fecal microbiota in 14 stool samples among laboratory-confirmed and non-confirmed infant botulism cases.

**Results:**

Seven bacterial phyla were identified among all 14 infant stool samples examined. Compared to samples from non-confirmed cases, the fecal microbiota of infant botulism patients displayed significantly higher *Proteobacteria* abundance. Of the 20 bacterial families identified, *Enterobacteriaceae* was significantly more abundant in samples from infants with botulism. *Firmicutes* abundance and the abundance ratio of *Firmicutes*/*Proteobacteria* was significantly lower in samples from infants with botulism. *Lactobacillus* spp. abundance was notably reduced in 12 of the 14 samples. *Clostridium botulinum* and *Clostridium baratii* were identified in low relative abundances in confirmed and non-confirmed samples based on their 16S rRNA gene profiles, although their toxigenicity remained undetermined. No significant differences were observed in the number of operational taxonomic units (OTUs) observed or in fecal microbiota diversity between laboratory-confirmed and non-confirmed samples. Correlations between individual phylum abundances and infant age were variable, and no significant differences were shown in number of OTUs observed or in fecal microbiota diversity between samples delineated by overall mean age.

**Conclusions:**

Significant differences in *Proteobacteria*, *Firmicutes*, and *Enterobacteriaceae* abundances were identified in the fecal microbiota of infants with botulism when compared to samples from non-confirmed cases. Fecal microbiota diversity was not significantly altered in infants with botulism, and a limited presence of BTPC was shown. It could not be determined whether the fecal microbiota profiles shown here were comparable prior to patient illness, or whether they were the direct result of infant botulism. The results of this study do, however, provide a detailed and descriptive observation into the infant gut microbiota after intestinal colonization by BTPC.

## Background

From 2001 to 2012, the National Botulism Surveillance System reported an average of 142 cases per year of botulism in the USA [1]. Infant botulism is responsible for 68.5 % of these cases. Each year, the National Botulism Laboratory Team (NBLT) at the Centers for Disease Control and Prevention (CDC) investigates approximately 20 suspected cases of infant botulism submitted by states across the USA. First described in 1976 [2], infant botulism occurs in children less than 1 year old, and infant susceptibility to gut colonization of botulinum neurotoxin (BoNT) producing clostridia (BTPC) may be associated with perturbations of the developing infant gut microbiota [3]. Strains of *Clostridium botulinum*, *Clostridium baratii*, and *Clostridium butyricum* have each been implicated as the causative agents in infant botulism cases. Positive confirmation of infant botulism is established when BoNT and/or isolates of BTPC are detected in the stool.

Compared to adults, infants harbor an intestinal microbiota (i.e., the assemblage of microorganisms which occupy the gut) that is transient and minimally complex [4, 5]. A number of factors are known to influence the microbial composition within the infant gut including age [6–8], mode of delivery [9, 10], diet [11–13], antibiotic use [14–16], and disease [17, 18], making it difficult to delineate their respective effects. An important function of the microbiota beyond digestion and metabolism is to prevent the propagation of pathogenic microorganisms [19–21]. It has been suggested that alterations of the developing infant gut microbiota may suppress BTPC colonization resistance, thus increasing susceptibility to botulism in certain individuals. Although studies using mouse models appear to support this claim, the etiology of this process remains unclear [22, 23].

Some risk factors identified for infant botulism include honey consumption, breastfeeding status, and living in a rural area [24–28]. Many of these risk factors have been shown to independently influence the microbiota in both healthy and diseased infants. However, delineating the degree of influence of botulism on the infant gut microbiota from disease-independent factors is a difficult challenge and not the focus of this study. Our aim was to provide a detailed descriptive analysis of the fecal microbiota of infants with botulism and to identify any significant alterations in bacterial abundance, composition, and/or diversity among samples from confirmed and non-confirmed infant botulism cases. To characterize the prokaryotic members of the infant gut microbiota, we targeted the hypervariable V3 region of the 16S rRNA gene for high-throughput sequencing and performed extensive downstream taxonomic analysis. This methodological approach allowed for a thorough and descriptive taxonomic characterization of the patient fecal microbiota and provided a means to evaluate disparities between the gut microbiota of infants with botulism and the gut microbiota of infants from non-confirmed botulism cases.

## Results

### Evaluation of amplification and sequencing bias

**Fig. 1:**
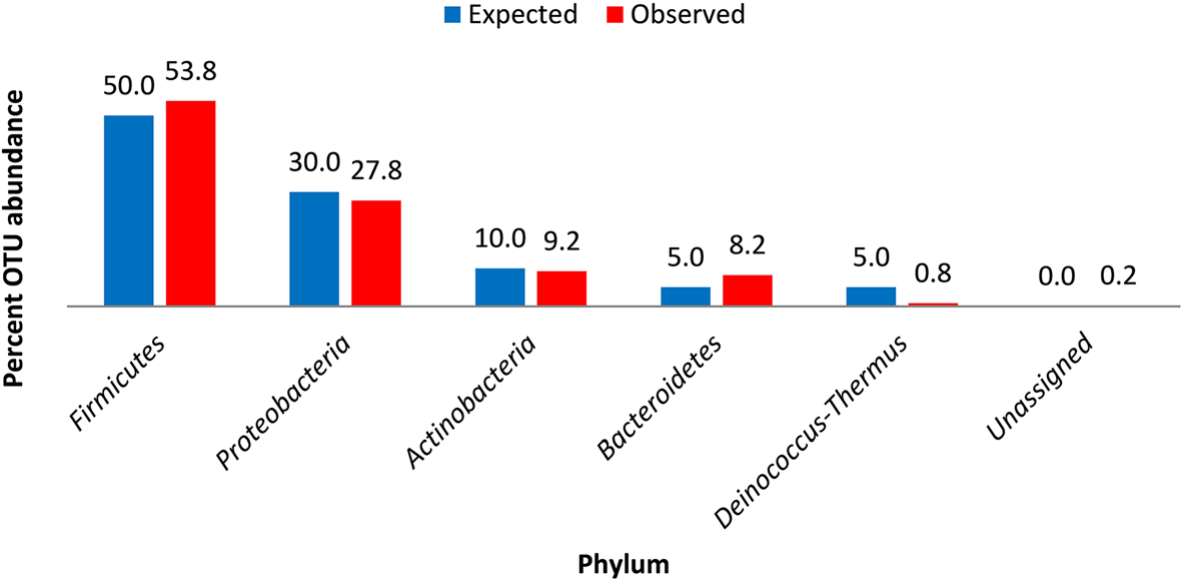
Histograms of mock community DNA-sequencing results. Expected phylum abundance percentages were calculated by classifying each of the 20 known bacterial species into their respective phylum. Observed phylum abundance percentages were calculated by assigning 16S rRNA gene sequence reads to representative taxonomy by alignment with Greengenes ribosomal database using an OTU definition of 97 % sequence homology or greater with a minimum cluster size of 3

Each of the five phyla from the mock community sample were successfully identified from sequence read analysis (Fig. 1). Overall mean absolute percent error (MAPE) for abundance quantification of the microbial mock community was 14.4 % (SD ± 33.1). Overall error was heavily skewed (MAPE = 74 %; SD ± 10) toward two phyla (*Bacteroidetes* and *Deinococcus-Thermus*), which represented the bottom 10 % of total phylum abundance. Abundance quantification of the top 90 % of phyla resulted in a MAPE of 7.6 % (SD ± 0.27). Results from mock community sequencing indicated that the study methodology employed was largely effective for phylum-level taxonomic analysis; however, the percent error may increase when quantifying underrepresented phyla.

### Sample and patient characteristics

We analyzed 14 infant stool samples from 14 patients representing a broad range of ages. Eight samples were associated with laboratory-confirmed botulism cases representing BoNT serotypes A, B, and F (designated as “confirmed”), and six samples were from cases where no laboratory confirmation of botulism could be made (designated as “non-confirmed”). Mean patient age across all 14 samples was 87.9 days (SD ± 98.3). Mean patient age in confirmed samples was 68.0 days (SD ± 88.7), and mean patient age in non-confirmed samples was 114.3 days (SD ± 120.8). There was no significant difference in patient age between confirmed and non-confirmed samples (*p* = 0.22; alpha = 0.05). Coded sample names, BoNT type detected, quantitative PCR results, and patient ages at the time of sample collection are listed in Table 1. No additional patient or sample metadata was available for these cases.

**Table 1:** Patient characteristics and sample properties of the 14 stool samples examined for this study

Stool	Sample	Patient age (days)	BoNT type^a^	PCR result
1	CDC68129	14	Type B	*bont/B*
2	CDC68058	16	Type F	*bont/F*
3	CDC69068	21	Type B	*bont/B*
4	CDC68083	25	Type A^b^	*bont/A bont/B*
5	CDC68116	28	Type A	*bont/A*
6	CDC68192	50	Type B	*bont/B*
7	CDC68090	120	Type B	*bont/B*
8	CDC68126	270	Type B	*bont/B*
9	CDC68053	17	None	Negative
10	CDC69114	46	None	Negative
11	CDC68112	63	None	Negative
12	CDC68122	90	None	Negative
13	CDC69062	120	None	Negative
14	CDC69078	350	None	Negative

^a^BoNT type refers to the botulinum toxin type detected in each corresponding sample via mouse bioassay

^b^In this case, the isolate of BTPC not only produced BoNT serotype A, but also harbored an unexpressed gene encoding *bont/*B

### Taxonomic and statistical analysis of fecal microflora

Sequence read statistics and alpha diversity metrics from 16S rRNA gene sequencing are listed in Table 2. The number of operational taxonomic units observed (OTU; defined as a subset of 3 or more sequence reads that share 97 % or greater sequence homology) was lowest for CDC68053 and highest for CDC68083 with an average number of OTUs per sample of 902.4 (SD ± 502.0). There was no significant difference in number of OTUs observed between confirmed and non-confirmed samples (*p* = 0.61; alpha = 0.05). Shannon diversity index, a measure of bacterial richness and evenness, revealed that the fecal microbiota from sample CDC68083 was highest in bacterial diversity while sample CDC68116 displayed lowest diversity. Fecal microbiota diversity was not significantly different between confirmed and non-confirmed samples (*p* = 0.52; alpha = 0.05).

**Table 2:** Sequence read statistics and alpha diversity metrics for each of the 14 stool samples examined for this study

Botulism	Sample	Total number of reads^a^	Number of reads passing quality filtering^a^	Number of OTUs observed^a^	Shannon diversity index^a^
Confirmed	CDC68129	747314	460195	496	2.3
	CDC68058	796510	376891	850	2.9
	CDC69068	706703	440769	637	2.5
	CDC68083	720976	510882	**2242**	**4.4**
	CDC68116	760653	543541	475	**0.7**
	CDC68192	**864853**	446933	928	3.4
	CDC68090	589121	369066	1615	3.2
	CDC68126	**496815**	396233	994	3.0
Non-confirmed	CDC68053	595735	433809	**418**	1.5
	CDC69114	723322	496246	517	2.4
	CDC68112	715786	**285335**	670	2.2
	CDC68122	831044	**602856**	676	2.1
	CDC69078	619394	392261	1117	3.8
	CDC69062	819457	412327	998	3.4

^a^Highest and lowest value in each column recorded in bold

**Fig. 2:**
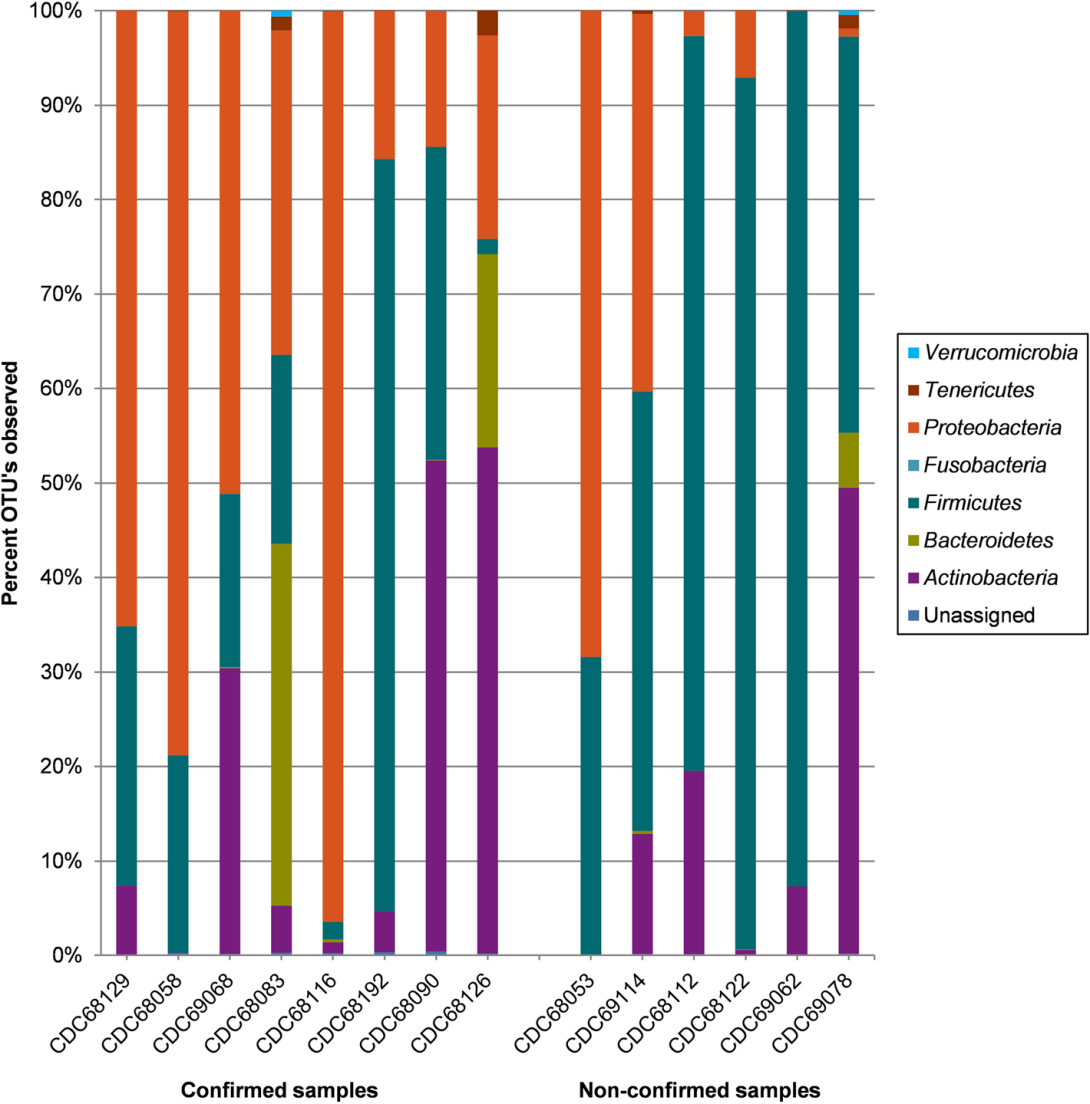
Stacked histograms illustrating phylum-level bacterial composition of the 14 stool samples. Ion Torrent 16S rRNA gene sequence reads were assigned to representative taxonomy by alignment with Greengenes ribosomal database using an OTU definition of 97 % sequence homology or greater with a minimum cluster size of 3

**Fig. 3:**
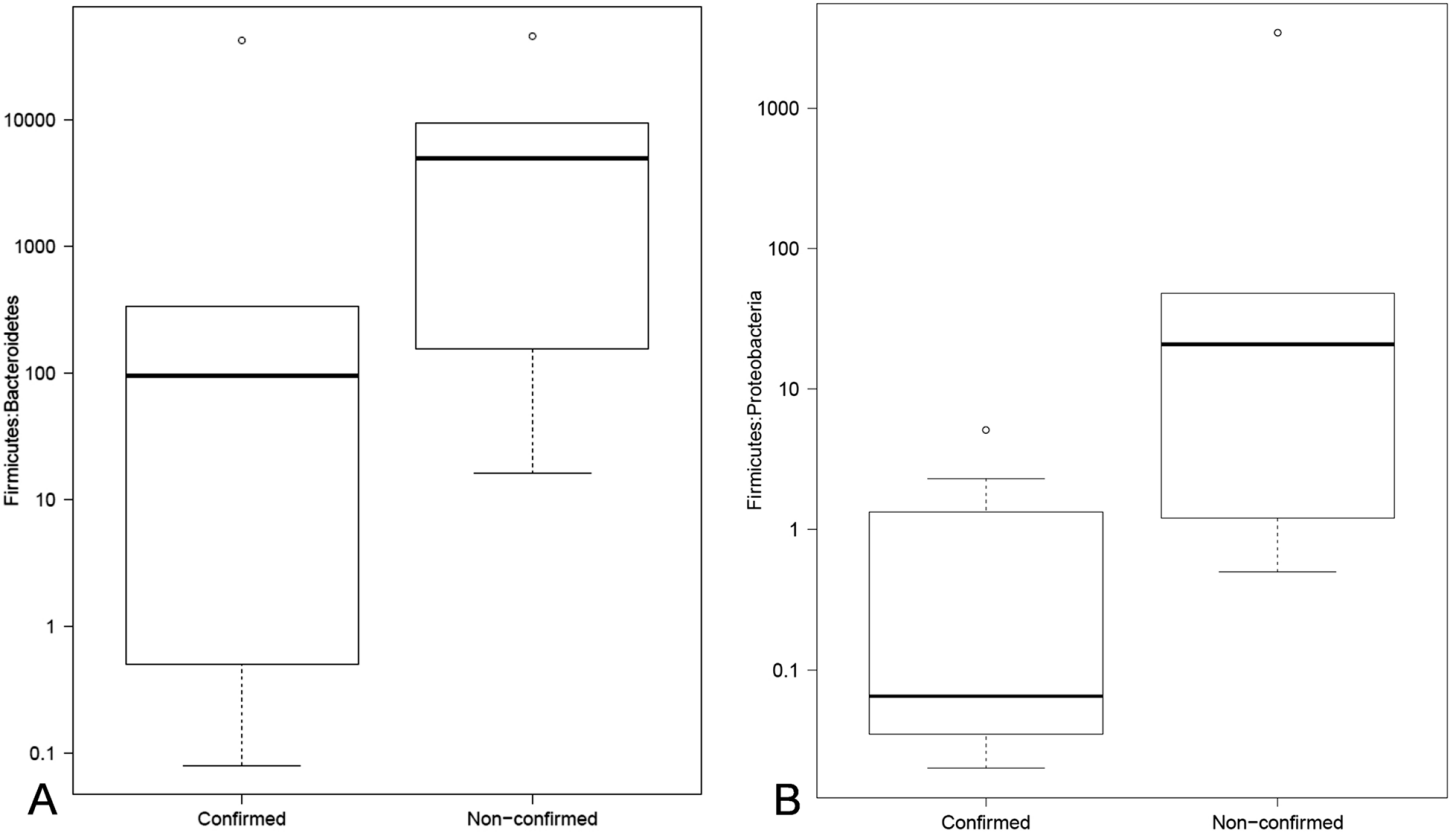
*Box plots* of the ratios of *Firmicutes*/*Bacteroidetes* (**a)** and *Firmicutes*/*Proteobacteria* (**b**) in confirmed and non-confirmed samples. Abundance measurements based on 16S rRNA sequence reads and assigned by alignment with Greengenes ribosomal database using an OTU definition of 97 % sequence homology with a minimum cluster size of 3. Outliers within each group are indicated by the *circular points* on the plots

**Fig. 4:**
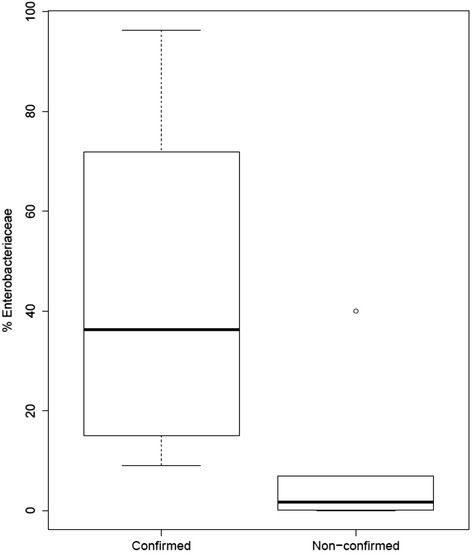
*Box plot* indicating the percent abundance of the bacterial family *Enterobacteriaceae* in confirmed and non-confirmed samples. Abundance measurements based on 16S rRNA sequence reads and assigned by alignment with Greengenes ribosomal database using an OTU definition of 97 % sequence homology with a minimum cluster size of 3. A single outlier within the non-confirmed group is indicated by the *circular point* on the plot

Phylum-level taxonomic analysis of the fecal microflora revealed the presence of 7 bacterial phyla which included *Firmicutes*, *Proteobacteria*, *Actinobacteria*, *Bacteroidetes*, *Tenericutes*, *Verrucomicrobia*, and *Fusobacteria* (Fig. 2). A small percentage of sequence reads (range = 0.02–0.39 %) could not be assigned to a phylum and therefore designated as “Unassigned”. *Firmicutes* percent abundance was significantly lower (*p* = 0.01; alpha = 0.05) in the fecal microbiota of botulism-confirmed samples (x̅ = 25.4 %; SD ± 24.6) compared to non-confirmed samples (x̅ = 63.8 %; SD ± 27.0), and *Proteobacteria* abundance was significantly higher (*p* = 0.01; alpha = 0.05) in confirmed samples (x̅ = 47.6 %; SD ± 31.1) compared to non-confirmed samples (x̅ = 8.6 %; SD ± 15.6). No other phylum displayed a significant difference in abundance between confirmed and non-confirmed samples. The ratio of *Firmicutes*/*Proteobacteria* was 515 times lower (*p* = 0.01, alpha = 0.05) in confirmed samples (x̅ = 1.1; SD ± 1.7) than in non-confirmed samples (x̅ = 586.6; SD ± 1392.2) (Fig. 3), and the ratio of *Firmicutes*/*Bacteroidetes* was 1.4 times lower (*p* = 0.11, alpha = 0.05) in confirmed samples (x̅ = 7292.6; SD ± 17459.2) than in non-confirmed samples (x̅ = 10563.8; SD ± 16825.8) (Fig. 4).

Family-level taxonomic analysis of the fecal microflora in the infant stool samples revealed the presence of 20 bacterial families (Table 3). Sequence reads that could not be assigned to a bacterial family, and families with <1.0 % abundance in each of the 14 samples, were designated as “Other”. The *Gammaproteobacteria* family *Enterobacteriaceae* displayed significantly higher abundance (*p* = 0.02, alpha = 0.05) in confirmed samples (x̅ = 44.0 %; SD ± 33.5) compared to non-confirmed samples (x̅ = 8.4 %; SD ± 15.7) (Fig. 3). No other bacterial family displayed a significant difference in abundance between confirmed and non-confirmed samples.

**Table 3:** Family-level percent bacterial composition for each of the 14 stool samples examined in this study

		Confirmed samples ^b^								Non-confirmed samples^b^					
Phylum	Family	CDC68129	CDC68058	CDC69068	CDC68083	CDC68116	CDC68192	CDC68090	CDC68126	CDC68053	CDC69114	CDC68112	CDC68122	CDC69062	CDC69078
*Actinobacteria*	*Actinomycetaceae*	0.0 %	0.0 %	0.1 %	0.0 %	1.0 %	2.4 %	0.0 %	0.0 %	0.0 %	0.0 %	0.0 %	0.4 %	0.0 %	0.0 %
	*Bifidobacteriaceae*	6.1 %	0.0 %	24.6 %	4.6 %	0.0 %	0.0 %	**51.9 %**	**53.5 %**	0.0 %	0.0 %	19.3 %	0.0 %	6.7 %	**48.2 %**
	*Coriobacteriaceae*	1.2 %	0.0 %	5.4 %	0.2 %	0.1 %	1.6 %	0.0 %	0.0 %	0.0 %	12.7 %	0.0 %	0.0 %	0.5 %	0.8 %
*Bacteroidetes*	*Bacteroidaceae*	0.0 %	0.0 %	0.1 %	**21.4 %**	0.3 %	0.0 %	0.0 %	20.5 %	0.0 %	0.3 %	0.0 %	0.0 %	0.0 %	1.4 %
	*Rikenellaceae*	0.0 %	0.0 %	0.0 %	0.0 %	0.0 %	0.0 %	0.0 %	0.0 %	0.0 %	0.0 %	0.0 %	0.0 %	0.0 %	4.2 %
	*Sphingobacteriaceae*	0.0 %	0.0 %	0.0 %	15.7 %	0.0 %	0.0 %	0.0 %	0.0 %	0.0 %	0.0 %	0.0 %	0.0 %	0.0 %	0.0 %
*Firmicutes*	*Paenibacillaceae*	0.0 %	0.0 %	0.0 %	0.0 %	0.0 %	0.0 %	0.0 %	0.0 %	0.0 %	0.0 %	0.0 %	0.0 %	3.7 %	0.0 %
	*Planococcaceae*	0.0 %	0.0 %	0.0 %	0.0 %	0.0 %	0.0 %	21.0 %	0.0 %	0.0 %	0.0 %	0.0 %	0.0 %	**40.2 %**	0.0 %
	*Enterococcaceae*	22.3 %	20.4 %	14.8 %	19.2 %	0.9 %	**42.8 %**	7.1 %	0.4 %	0.0 %	10.0 %	**72.9 %**	8.9 %	34.0 %	0.1 %
	*Lactobacillaceae*	0.0 %	0.0 %	0.0 %	0.0 %	0.3 %	1.3 %	0.0 %	0.0 %	31.4 %	0.0 %	0.6 %	**69.6 %**	0.0 %	0.0 %
	*Clostridiaceae*	3.5 %	0.1 %	0.3 %	0.1 %	0.1 %	11.9 %	4.1 %	0.2 %	0.0 %	35.1 %	0.0 %	4.4 %	0.5 %	0.8 %
	*ClostridialesFamilyXI*	0.0 %	0.0 %	3.2 %	0.0 %	0.2 %	0.4 %	0.2 %	0.0 %	0.0 %	0.0 %	0.0 %	0.0 %	12.1 %	0.0 %
	*Lachnospiraceae*	0.2 %	0.0 %	0.0 %	0.2 %	0.1 %	23.0 %	0.0 %	0.8 %	0.0 %	1.1 %	0.0 %	8.3 %	1.1 %	36.1 %
	*Ruminococcaceae*	1.3 %	0.0 %	0.0 %	0.4 %	0.1 %	0.2 %	0.0 %	0.2 %	0.0 %	0.2 %	0.0 %	0.0 %	0.5 %	3.5 %
*Proteobacteria*	*Sphingomonadaceae*	0.0 %	0.0 %	0.0 %	2.7 %	0.0 %	0.0 %	0.0 %	0.0 %	0.0 %	0.0 %	0.0 %	0.0 %	0.0 %	0.0 %
	*Comamonadaceae*	0.0 %	0.0 %	0.0 %	18.4 %	0.0 %	0.0 %	0.0 %	0.0 %	0.0 %	0.0 %	0.0 %	0.2 %	0.0 %	0.0 %
	*Desulfovibrionaceae*	0.0 %	0.0 %	0.0 %	4.1 %	0.0 %	0.0 %	0.0 %	0.0 %	0.0 %	0.0 %	0.0 %	0.0 %	0.0 %	0.0 %
	*Enterobacteriaceae*	**65.2 %**	**78.7 %**	**51.1 %**	9.1 %	**96.3 %**	15.7 %	14.4 %	21.5 %	0.1 %	**40.0 %**	2.7 %	6.9 %	0.0 %	0.8 %
	*Pseudomonadaceae*	0.0 %	0.0 %	0.0 %	0.0 %	0.0 %	0.0 %	0.0 %	0.0 %	**68.1 %**	0.0 %	0.0 %	0.0 %	0.0 %	0.0 %
*Tenericutes*	*Erysipelotrichaceae*	0.0 %	0.1 %	0.0 %	1.5 %	0.1 %	0.0 %	0.0 %	2.6 %	0.0 %	0.3 %	0.0 %	0.0 %	0.0 %	1.5 %
Other^a^		0.1 %	0.7 %	0.5 %	2.4 %	0.5 %	0.8 %	1.2 %	0.2 %	0.4 %	0.3 %	4.3 %	1.2 %	0.7 %	2.6 %

^a^Includes family-level taxa that were either unassigned or represented <1.0 % abundance in each of the 14 samples

^b^Highest percent abundance in each column recorded in bold

Based on 16S rRNA gene taxonomy, *C. botulinum* was identified in 6 of the 8 confirmed samples and 2 of the 6 non-confirmed samples, with relative abundances ranging from <0.001 to 0.01 % of the overall bacterial community where detected (Table 4). *C. baratii* was identified in 5 of the 8 confirmed samples and 2 of the 6 non-confirmed samples with relative abundances ranging from <0.001 to 0.003 % of the overall bacterial community where detected (Table 4).

**Table 4:** Percent *Clostridium botulinum* and *Clostridium baratii* 16S rRNA gene sequences observed for each sample

Botulism	Sample	*Clostridium botulinum* (%)	*Clostridium baratii* (%)
Confirmed	CDC68129	0.0	9.4 × 10^−4^
	CDC68058	7.3 × 10^−6^	8.6 × 10^−4^
	CDC69068	0.0	0.0
	CDC68083	6.7 × 10^−5^	0.0
	CDC68116	5.8 × 10^−5^	4.5 × 10^−6^
	CDC68192	3.4 × 10^−3^	3.2 × 10^−3^
	CDC68090	1.0 × 10^−2^	0.0
	CDC68126	1.1 × 10^−4^	2.7 × 10^−6^
Non-confirmed	CDC68053	0.0	5.7 × 10^−6^
	CDC69114	0.0	0.0
	CDC68112	1.7 × 10^−6^	0.0
	CDC68122	2.3 × 10^−5^	6.3 × 10^−4^
	CDC69078	0.0	0.0
	CDC69062	0.0	0.0

**Fig. 5:**
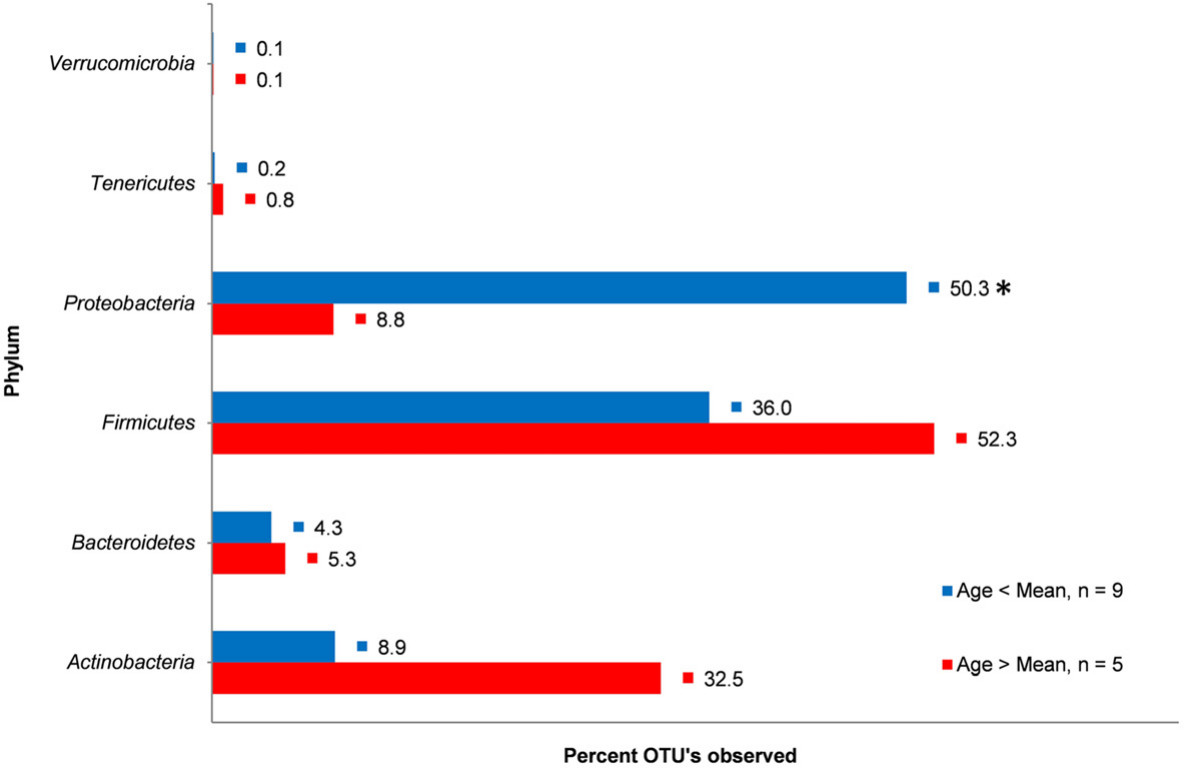
Phylum-level abundance percentages relative to age. Abundance measurements based on 16S rRNA gene sequence reads and assigned by alignment with Greengenes ribosomal database using an OTU definition of 97 % sequence homology with a minimum cluster size of 3. Samples representing infants younger than the overall mean infant age of 87.9 days include CDC68129, CDC68058, CDC9068, CDC68083, CDC68116, CDC68192, CDC68053, CDC69114, and CDC68112, and samples representing infants older than the overall mean infant age of 87.9 days include CDC68090, CDC68126, CDC68122, CDC69062, and CDC69078. Ages are based on the age of the infant at the time of sample collection. The *asterisk* indicates a significant difference in phylum abundance was calculated between the two age groups

Samples were re-grouped relative to mean patient age of all 14 samples to examine the association between age and phylum-level abundance. Nine samples were collected from patients younger than the overall mean patient age (collectively referred to as the “younger” group), and 5 samples were collected from patients older than the overall mean patient age (collectively referred to as the “older” group). *Proteobacteria* abundance was significantly higher (*p* = 0.04; alpha = 0.05) among the younger group (x̅ = 50.3 %; SD ± 28.5) compared to the older group (x̅ = 8.8 %; SD ± 8.2) (Fig. 5). No other phylum displayed a significant difference in abundance between the younger and older group. The average ratio of *Firmicutes*/*Proteobacteria* was 170 times lower (*p* = 0.13; alpha = 0.05) in the younger group (x̅ = 4.1; SD ± 9.3) compared to the older group (x̅ = 698.3; SD ± 1526.2), and the average ratio of *Firmicutes*/*Bacteroidetes* was 6.0 times lower (*p* = 0.57; alpha = 0.05) in the younger group (x̅ = 13671.8; SD ± 20643) compared to the older group (x̅ = 2287.1; SD ± 3028.2) (data not shown). There was no significant difference (*p* = 0.28; alpha = 0.05) in number of OTUs observed between the younger infant group and the older infant group. Fecal microbiota diversity was not significantly different between the younger infant group and the older infant group (*p* = 0.20; alpha = 0.05).

**Fig. 6:**
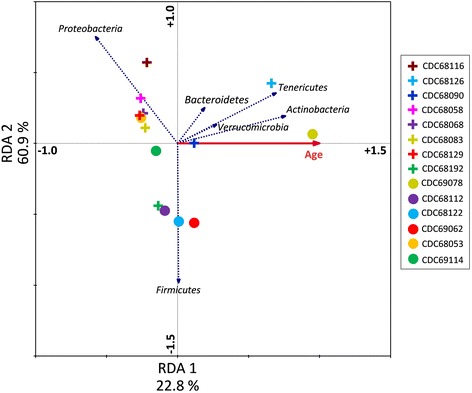
RDA ordination diagram displaying the associations between samples, phylum abundance, and age. *Crosses* represent confirmed samples, and *circles* represent non-confirmed samples. The *solid red* vector was generated from age data representing the age of the infant at the time of sample collection, and *blue* vectors represent phylum-level abundances. Each vector points in the direction of steepest increase of value, and the length of each vector indicates the measure of fit for each sample relative to the vector. Angles between vectors indicate the correlation between the individual factors. Approximate correlations are positive when the angle is <90° and negative when the angle is >90°

Redundancy analysis (RDA) was used to explore associations among infant microbiota composition and abundance, and to examine the influence of infant age on sample variance (Fig. 6) [29]. Taxonomically unclassified data were excluded from RDA analysis, as was data representing the phylum *Fusobacteria* which was only detected in trace abundance (<0.002 %) in one sample. RDA explained 83.7 % of all variation among samples. Overall sample variance was produced by the combined effect of bacterial abundance, bacterial composition, and infant age (*F* = 3.5; *p* = 0.048; alpha = 0.05). RDA confirmed a negative correlation between *Proteobacteria* abundance and infant age, and positive correlations between infant age and *Tenericutes*, *Actinobacteria. Bacteroidetes*, and *Verrucomicrobia* abundance. RDA revealed that *Proteobacteria* abundance was positively correlated with 7 samples (CDC68116, CDC68058, CDC68068, CDC68129, CDC68053, CDC68083, and CDC69114). Three samples (CDC68126, CDC69078, and CDC68090) were positively correlated to *Tenericutes*, *Actinobacteria*, *Verrumicrobia*, and to a lesser extent *Bacteroidetes* abundance. The phylum *Firmicutes* displayed a negative correlation to all other phyla by RDA, and displayed a positive correlation with samples CDC69062, CDC68122, CDC68112, and CDC68192. The influence of age was weighted most heavily on sample CDC69078 which was 7.2 times greater than its effect on all other samples.

## Discussion

Characterizing the fecal microbiota in infants with botulism provides another step toward understanding the link between the infant gut microbiota and this disease. Although enteric diseases can produce discernable, if not predictable, alterations to the bacterial gut microbiota [30], it can also be influenced by a wide variety of disease-independent perturbations [31]. The findings of our study revealed the fecal microbiota of infants with botulism was characterized by an over-enrichment of *Proteobacteria* with low *Firmicutes* abundance. This finding highlighted a significant disparity between confirmed and non-confirmed samples with a clear deviation from a more traditional healthy-infant microbiota profile [16]. Previous studies have reported reduced *Firmicutes* abundance with a concomitant increase in *Proteobacteria* abundance in response to disease-mediated alterations of the gut microflora [32, 33]. *Firmicutes* are often highly represented in the gut microbiota of healthy individuals and can be reduced in illness [34], while a significant increase in *Proteobacteria* abundance can lead to gastrointestinal inflammation in response to environmental and genetic factors [35].

A notable disparity was identified among the relative proportions of *Lactobacillus* spp. identified among samples. The genus *Lactobacillus* contains over 180 species of bacteria, many of which are commonly found propagating the microbiota of healthy infants. *Lactobacillus* spp. abundance was markedly reduced (mean relative abundance = 0.2 %; SD ± 0.004) in the fecal microbiota of all 8 confirmed samples, and 4 of the 6 non-confirmed samples (data not shown). Similar reductions have been reported in previous studies investigating the fecal microbiota of infants [5, 36–38]. Among these reports, it was suggested that a variety of factors (e.g. caesarian delivery, aggressive antibiotic treatment, long-term incubation) may combine to suppress *Lactobacillus* colonization, significantly delay the establishment of beneficial bacteria, and increase susceptibility to enteric pathogen colonization. Lactobacilli have been shown to provide some inhibition activity against several common food pathogens [39]. Although it remains unclear whether the reduction of beneficial bacteria such as *Lactobacillus* spp. can increase susceptibility to BTPC colonization in infants, this finding illustrates a distinct feature of the gut microbiota profile in infants once BTPC colonization has been established.

Mariat et al. [40] illustrated the utility of using phylum-level abundance ratios to profile the infant gut microbiota. In that study, it was reported that the ratio of *Firmicutes*/*Bacteroidetes* in healthy infants was significantly lower than that of adults. Although we did not observe a significant difference in *Firmicutes*/*Bacteroidetes* abundance ratios between our sample groups, we do report a significant decrease in the abundance ratio of *Firmicutes*/*Proteobacteria* in confirmed infant botulism samples; a ratio which was 515-times lower than that observed in samples from non-confirmed cases. Whether this reduction preceded BTPC colonization, or whether it was a consequence of botulism could not be concluded. It should be noted that phylum abundances could have been influenced by gaps in sample collection (the time between onset of illness and sample collection) and sample storage times prior to DNA extractions. Nevertheless, taxonomic analysis indicated that the primary disparity between confirmed and non-confirmed samples resulted from significant differences in the relative abundances of *Firmicutes* and *Proteobacteria* within the fecal microbiota.

Only a handful of studies have reported their efforts to characterize the fecal microflora in infants with botulism. Long et al. (1985) [27] examined stool samples from seven infant botulism patients and found a high proportion of enterobacteria in the fecal microflora. Similarly, we report significantly higher *Enterobacteriaceae* abundance in the fecal microflora of infants with botulism compared to samples from non-confirmed cases. A comparison of family-level abundances revealed *Enterobacteriaceae* to be the only bacterial family which displayed a significant disparity between confirmed and non-confirmed samples. *Enterobacteriaceae* was the most abundant bacterial family identified in the fecal microbiota of infants with botulism averaging 44.0 % of total bacterial family abundance. In contrast, *Enterobacteriaceae* abundance in non-confirmed samples averaged only 8.4 % of all bacterial families identified, making it the fifth most abundant family within that group. This disparity is not surprising as this family of *Gammaproteobacteria* contains many gut pathogens which are responsible for a variety of gastrointestinal illnesses and can flourish in the gastrointestinal tract of infants with systemic infections [41–43]. Long et al. suggested that an enterobacteria-enriched infant gut may reflect a transitional stage of development where susceptibility to *C. botulinum* colonization is increased. This transition may be driven by dietary changes among the infants, or due to factors that have yet to be determined. Nonetheless, these findings could indicate a possible link between infant botulism and *Enterobacteriaceae* colonization; however, with only a limited number of studies available for comparison, follow-up investigations would be required to establish a clear trend.

In another study, Wilcke et al. [26] quantified *C. botulinum* from stool samples of four infants with laboratory-confirmed infant botulism and found that *C. botulinum* abundance ranged from 0.01 to 3.3 % of culturable fecal microbiota. In our study, neither C*. botulinum* nor *C. baratii* ever exceeded 0.01 % of the fecal microbiota. Although it is likely that BTPC intestinal abundance fluctuates temporally over the course of the illness, these findings do support the widely-held assumption that botulism can manifest from a relatively limited presence of BTPC in the infant gut. Unfortunately, there is no available data to indicate whether there is a minimum threshold for BTPC colonization needed to produce an amount of toxin required for disease onset. Given the extremely high potency of BoNT these organisms are capable of producing, coupled with the low weight of infants relative to adults, it was not surprising to find no more than 0.01 % BTPC abundance in stool samples from infant botulism patients.

The identification of *C. botulinum* and *C. baratii* 16S rRNA gene sequences in both botulism-confirmed and non-confirmed samples highlight the current limitations of using a sequencing-based approach for botulism diagnostics. Infant botulism is confirmed by the laboratory detection of BoNT and/or the detection of BTPC in infant stool. Although *C. baratii* can produce BoNT subtype F, non-toxigenic strains are more commonly isolated from stools. Non-toxigenic *C. baratii* do not harbor the serotype F botulinum neurotoxin gene (*bont*/F) within their genome, and as a result, 16S rRNA gene sequencing does not provide the necessary resolution to distinguish between toxigenic and non-toxigenic isolates of this organism. Similarly, amplicon sequencing cannot resolve *C. botulinum* from non-toxigenic strains of clostridia (such as *C. sporogenes*) that share >99 % 16S rRNA gene identity.

The temporal development of the infant gut microbiota throughout the first year of life is not consistent across all individuals [7, 8]. This yearlong progression from near sterility to a fully developed microbiome is guided by a myriad of external factors and can be interrupted by illness [6, 10, 44]. With the exception of *Proteobacteria* abundance, the microbiota in the younger infant group did not significantly differ from that of the older infant group in composition, abundance, or diversity. RDA indicates a negative correlation between infant age and samples with higher *Proteobacteria* abundance, and a positive correlation between infant age and samples with higher abundances of *Actinobacteria*, *Tenericutes*, *Verrumicrobia*, and *Bacteroidetes*. Excluding age as a co-variable in RDA (data not shown) did not alter the cluster patterns shown in Fig. 6, nor the correlations approximated between samples and phylum composition and abundance. Together, these findings indicate that differences in fecal microbiota abundance and composition between botulism-confirmed and non-confirmed samples were not due solely to patient age disparities.

## Conclusions

The infant gut microbiota undergoes a dramatic transition throughout the first year of life, and a variety of factors guide its development. Perturbations of the infant gut microbiota have been shown to increase susceptibility to opportunistic pathogens, yet it remains unclear whether similar disturbances can be associated with increased susceptibility to BTPC colonization. Researchers have speculated that infants display an increased susceptibility to botulism due to their transient and minimally complex gut microbiota; however, there is little published data to support this claim. In an effort to resolve some of the uncertainty surrounding this speculation, we conducted the first detailed analysis into the gut microbiota of infants with botulism using high-throughput, next-generation sequencing technology.

We have identified several distinct features among BTPC-colonized microbiota which are comparable to those previously identified in infants suffering from botulism and other enteric diseases. Our findings revealed that, compared to that of non-confirmed samples, the fecal microbiota of infants with botulism contained significantly higher abundances of *Proteobacteria* and *Enterobacteriaceae*, while *Firmicutes* abundance remained significantly lower. Additionally, our findings indicated that botulinum toxin-producing clostridia were present in very low abundances, indicating that botulism can manifest from a relatively limited presence of these organisms in the infant gut.

These findings can be used to guide future infant botulism research efforts by directing focus toward several distinct features of the infant gut microbiota which have been identified here. The data generated from this study has greatly expanded our insight into the gut microbiota profiles in infant botulism patients. Ultimately, our understanding of infant botulism will continue to increase as sequencing technologies and computational analyses are further developed. These developments should greatly increase our capabilities as investigators interested in gaining a deeper understanding of botulism and its influence on children impacted by this disease.

## Methods

### Sample properties and patient characteristics

In compliance with a human subjects exemption protocol (#4991.0) approved by the CDC Human Research Protection Office, coded stool samples were selected for investigation from 14 infants suspected to have botulism with patient ages ranging from 14 to 350 days old. The samples were selected to represent a broad array of sample properties and patient characteristics. Of the 14 samples sent for botulism testing, NBLT confirmed 8 samples for infant botulism (confirmed), while 6 could not be confirmed (non-confirmed). Refer to Table 1 for complete sample properties and patient characteristics.

### Genomic DNA extraction and sequencing

Genomic DNA was extracted from each stool sample using the PowerFood^®^ Microbial DNA Isolation Kit (MO BIO Laboratories, Inc., Carlsbad, CA). Libraries were prepared for Ion Torrent PGM sequencing following the Fusion Method—Ion Amplicon Library Preparation for bidirectional sequencing (Ion Amplicon Library Preparation [Fusion Method] User Guide; Publication number 4468326, Revision C). Extracted gDNA was purified using the DNA Clean and Concentrator-5 kit (Zymo Research, Irvine, CA) and quantified on a NanoDrop 2000 UV–vis Spectrophotometer (Thermo Scientific, Wilmington, DE). For bidirectional sequencing, two direct PCR amplifications targeting the hypervariable V3 region of the 16S rRNA gene were performed on each sample gDNA. PCR target primers were previously described by Zhang et al. [45] and synthesized with Ion Torrent compatible adaptors and key sequences to generate approximately 280 bp PCR amplicons (Table 5). Each 50 μL PCR reaction consisted of 45 μL of Platinum PCR SuperMix High Fidelity buffer (Life Technologies, Grand Island, NY), 1 μL each of 10 μM primers and 50 ng of purified gDNA. PCR thermocycling conditions was set up with an initial denaturation at 94 °C for 2 min followed by 35 cycles of 94 °C for 30 s, 58 °C for 30 s, and 68 °C for 30 s with a final extension of 68 °C for 2 min. Following amplification, PCR products were purified using the DNA Clean and Concentrator-5 kit (Zymo Research), and size selection on the purified amplicons was performed using the E-Gel Agarose Gel Electrophoresis System (Life Technologies). Each size-selected library was quantified using the Qubit dsDNA HS Assay Kit (Life Technologies), diluted to 18 pM, and pooled into an equimolar solution. Each DNA fragment library was templated onto Ion Sphere Particles (ISPs) via emulsion PCR using the Ion OT2 instrument, and the resulting template-positive ISPs were quality checked to assess templating efficiency. Template-positive ISPs were enriched on the Ion ES instrument, loaded onto Ion 314 v2 chips, and sequenced using the Ion PGM.

**Table 5:** Primers used for 16S rRNA gene amplification of gDNA extracted from stools

Fusion PCR Primer	Sequencing adapters	Library key	16S rRNA Gene target region	Target name
A forward	5’-CCATCTCATCCCTGCGTGTCTCCGAC	TCAG	CCTACGGGAGGCAGCAG-3’	P1
trP1 forward	5’-CCTCTCTATGGGCAGTCGGTGAT	–	CCTACGGGAGGCAGCAG-3’	P1
A reverse	5’-CCATCTCATCCCTGCGTGTCTCCGAC	TCAG	ATTACCGCGGCTGCT-3’	P2
trP1 reverse	5’-CCTCTCTATGGGCAGTCGGTGAT	–	ATTACCGCGGCTGCT-3’	P2

Genomic DNA from Microbial Mock Community B (Even, High Concentration), v. 5.1H (bei Resources) was used to evaluate the effects of amplification and sequencing bias. The mock community sample of gDNA consisted of a single pool of equimolar RNA operon counts from 20 bacterial strains representing 5 bacterial phyla. Library preparation, sequencing, and taxonomic analysis of the mock community DNA was performed in the same manner as stool sample gDNA. A phylum-level comparison of actual abundance (known phylum percent distribution of the mock community) versus observed abundance (PGM sequencing results) was performed. The formula below was used to calculate mean absolute percentage error (MAPE) in abundance quantification (*F*_*t*_ = observed abundance, *A*_*t*_ = actual abundance, *n* = 5):

$$ MAPE=\frac{1}{n}\;{\displaystyle \sum_{t=t}^n\left|\frac{F_t-{A}_t}{A_t}\right|}\times 100 $$

Raw sequence data and the associated metadata for all 14 samples have been deposited in MG-RAST metagenomics analysis server (http://metagenomics.anl.gov). All sample data is freely available and openly accessible from the MG-RAST website under project number 13930, or by navigating directly to http://metagenomics.anl.gov/metagenomics.cgi?page=MetagenomeProject&project=13930.

### Sequence analysis and taxonomic characterization

Sequence reads were analyzed with the open source software package Quantitative Insights into Microbial Ecology v1.8.0 (QIIME) [46]. Prior to analysis, reads with a read length of less than 150 bp or greater than 350 bp, reads with missing quality scores or mean quality scores below 25, reads with primer mismatches, and reads with greater than 6 ambiguous bases were removed from analysis. The remaining reads were aligned using Python Nearest Alignment Space Determination (PyNAST), and clustered into operational taxonomic units (OTUs). OTU clustering is a metric of binning sequence reads where a single OTU is defined as a subset of reads (minimum cluster size of 3) which share 97 % or greater sequence homology. The QIIME workflow used for this analysis can be accessed via https://github.com/tbshirey/QIIME.

The representative taxonomic identities were assigned by alignment with the Greengenes ribosomal database [47], and stacked histograms of the relative taxonomic abundances were generated showing phylum-level taxonomic representatives for each sample. Family-level percent abundances, *Enterobacteriaceae* abundance, and abundance of two expected BTPC (*C. botulinum* and *C. baratii*) were also recorded for each sample. Phylum-level ratios of *Firmicutes*/*Proteobacteria* and *Firmicutes*/*Bacteroidetes* were calculated based on average abundance percentages for each sample. *Bacteroidetes* was not detected in samples CDC68129 and CDC68192 which prevented calculation of the *Firmicutes*/*Bacteroidetes* ratio for these samples.

### Statistical analyses

Significance testing was performed using the Mann-Whitney *U* rank-sum test in Statistical Package for the Social Sciences (SPSS; IBM Corporation, Armonk, NY). A non-parametric 0.95 confidence interval (*α* = 0.05) accompanied these estimates. Linear regression (reported as the adjusted *R*^2^ value; *R̅*^2^) was performed in SPSS to model the correlation between taxonomic abundance and infant age. Shannon diversity index illustrating inter-sample fecal microflora taxa diversity was calculated in QIIME by rarifying all samples. This index is a commonly used measure of diversity which uses an algorithm to account for both species richness (i.e., the number of species present) and species evenness (i.e., the distribution of species) within a sample. The Shannon diversity index estimates diversity on a scale beginning at 0 (no diversity observed) and increasing where greater diversity is estimated.

RDA was performed using the software package Canoco 4.5 (Microcomputer Power, Ithaca, New York) to examine the relationships between sample microbiota community structure, disease confirmation, and infant age. Bacterial phyla (dotted blue vectors) served as response variables, and patient age at the time of sample collection (solid red vector) was used as a predictor co-variable. Sample symbols were generated to indicate disease confirmation (crosses = botulism confirmed samples; circles = non-confirmed samples). Vector length is proportional to abundance (phylum vectors) or age (age vector), with each variable increasing in the direction of the vector. Correlations between samples and vectors are approximated based on sample proximity to each vector (indicating correlation between the sample and phylum composition and/or age), and the location of the sample point along the length of each vector (indicating correlation between the sample and phylum abundance, and/or age). Prior to ordination, Monte Carlo permutation tests (499 permutations under reduced model) were performed on non-transformed data. RDA was visualized using CanoDraw™ (Microcomputer Power). All other data visualized in tables, histograms, and bar charts were managed in Microsoft Excel (Microsoft, Redmond, WA), and box plots were generated using R (http://cran.r-project.org/).
